# Expanding the therapeutic landscape of minoxidil for androgenetic alopecia: topical, oral and sublingual formulations 

**DOI:** 10.3389/fphar.2025.1718208

**Published:** 2026-01-21

**Authors:** Flavia Rodrigues Dias, Shin Shen Yong, Holly FitzGerald, Rodney D. Sinclair, Bevin Bhoyrul

**Affiliations:** 1 SinclairDermatology, Melbourne, VIC, Australia; 2 Division of Dermatology, Department of Medicine, Universiti Malaya, Kuala Lumpur, Malaysia

**Keywords:** alopecia, androgenetic alopecia, minoxidil, topical minoxidil, oral minoxidil, low-dose oral minoxidil, sublingual minoxidil

## Abstract

Androgenetic alopecia (AGA) is the most common form of non-scarring alopecia, affecting up to 80% of men and 50% of women by the age of 70. Minoxidil, initially developed as an oral antihypertensive, has become one of the most widely used therapies for AGA due to its safety and efficacy. This review presents an overview of current evidence on topical, oral and sublingual minoxidil. Topical minoxidil, the only FDA-approved treatment for AGA in both men and women, promotes hair growth through several proposed mechanisms described in the article. Randomised controlled trials show that 5% formulations consistently increase hair counts, although results vary due to differences in follicular sulfotransferase activity. Low-dose oral minoxidil (0.25–5 mg) has emerged as a practical option for patients unresponsive to topical therapy. Hypertrichosis is the most frequent adverse effect, while cardiovascular events are uncommon at low doses. Sublingual administration, a novel delivery route that bypasses first-pass metabolism, may enhance follicular bioavailability while limiting systemic exposure. Early evidence indicates similar efficacy to oral therapy, with a potentially lower risk of cardiovascular effects. Overall, topical minoxidil remains first line, while oral and sublingual formulations expand therapeutic options and support individualised management. Further large-scale, long-term studies are needed to define optimal dosing, confirm safety and determine whether sublingual administration offers consistent advantages over oral use.

## 1 Introduction

Androgenetic alopecia (AGA) is the most common form of non-scarring alopecia globally, affecting up to 80% of men and 50% of women by the age of 70 (Norwood, 1975; Birch et al., 2001). It is a chronic, progressive condition characterised histopathologically by hair follicle (HF) miniaturisation resulting in the gradual conversion of terminal hairs into vellus-like hairs. In men, the typical presentation is bitemporal recession and vertex hair thinning (male pattern hair loss, MPHL), while women typically show diffuse thinning over the central scalp with retention of the frontal hairline (female pattern hair loss, FPHL) (Olsen, 2001; Hamilton, 1951).

AGA is a highly heritable trait with a likely polygenic mode of inheritance. Two major susceptibility loci implicated in AGA are the androgen receptor (AR)/ectodysplasin A2 receptor (EDA2R) locus on the X chromosome and a locus on chromosome 20p11, for which no candidate gene has yet been identified (Hillmer et al., 2005; Richards et al., 2008). The pathophysiology of MPHL is androgen-dependent, particularly involving dihydrotestosterone (DHT), which binds to androgen receptors in HF dermal papilla cells (DPCs). This interaction shortens the anagen phase, prolongs telogen and promotes miniaturisation of affected HFs, ultimately leading to a reduction in hair density and scalp coverage (Lolli et al., 2017). The involvement of androgens in the pathogenesis of FPHL is more complex and less well understood.

Although AGA is not life-threatening, its psychological impact is substantial. Studies have consistently demonstrated that AGA can result in impaired self-esteem, body image dissatisfaction, social anxiety and even depressive symptoms, especially among younger individuals (Cash, 1992; Hunt and McHale, 2005). The demand for effective and tolerable therapies for AGA continues to grow due to the condition’s high prevalence and psychosocial burden.

Currently, the only FDA-approved pharmacological treatments for AGA are topical minoxidil and oral finasteride. However, finasteride is approved for use in men only due to teratogenicity concerns in women of child-bearing age, and its use is associated with potential sexual and psychological side effects in a minority of users (Irwig, 2012; Kaufman, 2002). By contrast, topical minoxidil is approved for both sexes and remains the first-line therapy in most clinical scenarios due to its safety profile and established efficacy.

Minoxidil was initially developed as a potent oral antihypertensive medication. Hair growth stimulation was noted as a side effect in patients receiving oral minoxidil, which led to the development of a topical formulation for the treatment of AGA (Gupta and Charrette, 2015). Topical minoxidil was first approved for MPHL in 1988 (2% solution), followed by the 5% solution in 1991 and 5% foam in 2006. For women, the 2% solution was approved in 1991 and the 5% foam was subsequently approved in 2014 (Olsen et al., 2007). Even though it is now widely used, topical minoxidil is not without limitations. Adverse effects such as contact dermatitis, itching and unwanted facial hair growth may reduce compliance, and approximately 60% of users do not achieve visible improvements (Tosti et al., 2006; Blume-Peytavi et al., 2011).

In recent years, alternative systemic routes of administration of minoxidil, including oral and sublingual, have gained attention (Jimenez-Cauhe et al., 2020; Vano-Galvan et al., 2021; Bokhari et al., 2022). Low-dose oral minoxidil (LDOM), typically prescribed at doses ranging from 0.25 to 5 mg, has shown comparable or superior results to topical therapy in both men and women, including those individuals who previously failed topical treatment (Jimenez-Cauhe et al., 2020). The mechanism is presumed to be similar, although systemic exposure may lead to more consistent follicular activation. Sublingual administration, an emerging route that bypasses hepatic first-pass metabolism, may allow for even lower effective dosing and improved bioavailability, although current data are limited to early observational studies and case series (Bokhari et al., 2022).

This review provides a comprehensive evaluation of minoxidil use in AGA, focusing on its pharmacology, clinical efficacy, safety and tolerability across the three primary routes of administration: topical, oral and sublingual. We summarise evidence from randomised controlled trials (RCTs), observational studies and real-world clinical data to provide a critical appraisal of its role in AGA management.

## 2 Literature search

This review was conducted as a narrative synthesis to provide an up-to-date overview of the role of minoxidil in the treatment of AGA.

A comprehensive search of the literature was performed to identify relevant studies on the pharmacology, mechanisms of action, and clinical efficacy of topical, oral and sublingual minoxidil in the treatment of AGA. Searches were performed in PubMed (MEDLINE) and covered all available records up to 8 November 2025. The search strategy combined the following MeSH terms: “androgenetic alopecia”, “male pattern hair loss”, “female pattern hair loss”, “baldness, male pattern”, “baldness, female pattern”, “minoxidil” and “drug overdose”.

Filters were applied to include human, English-language studies published from 1980 to the present date. Eligible publication types included RCTs, cohort and case-control studies, meta-analyses, systematic reviews, observational studies, case reports, clinical practice guidelines, and pharmacological reviews.

Studies were included if they:

- Investigated minoxidil (any formulation or route) as a therapeutic intervention for AGA in adults;
- Reported clinical or mechanistic outcomes relevant to efficacy or safety;
- Were published in peer-reviewed journals.

Studies were excluded if they:

- Addressed alopecias other than AGA (e.g., alopecia areata, scarring alopecia);
- Described formulations unrelated to minoxidil;
- Were non-scientific summaries, editorials or conference abstracts without primary data.

After removing duplicates, a total of approximately 4855 records were identified through database searches. Following title and abstract screening, around 250 publications were selected for full-text review. Of these, 96 studies met all inclusion criteria and were included based on their relevance to this narrative review.

## 3 Pharmacokinetics

Minoxidil is a piperidine pyrimidine derivative with chemical structure 2,6-diamino-4-piperidinopyrimidine-1-oxide (C_9_H_15_N_5_O) (Rossi et al., 2012). Orally administered minoxidil is absorbed from the gastrointestinal tract at a rate of 95% and is metabolised extensively in the liver (Gupta et al., 2022a). Plasma levels of the parent drug peak within the first hour and decrease rapidly thereafter. Minoxidil does not bind to plasma proteins or cross the blood-brain barrier (Suchonwanit et al., 2019; Lowenthal and Affrime, 1980). Although the elimination half-life of minoxidil is 4.2 h (Kirkland, 2013; Pharmacia & Upjohn Company LLC, 2015), the hypotensive effect may persist for up to 72 h, suggesting extravascular accumulation (Canaday, 1980). The excretion of minoxidil and its metabolites predominantly occurs through the kidneys 3–4 h after administration (Sica, 2004).

## 4 Mechanism of action of minoxidil in androgenetic alopecia

**FIGURE 1:**
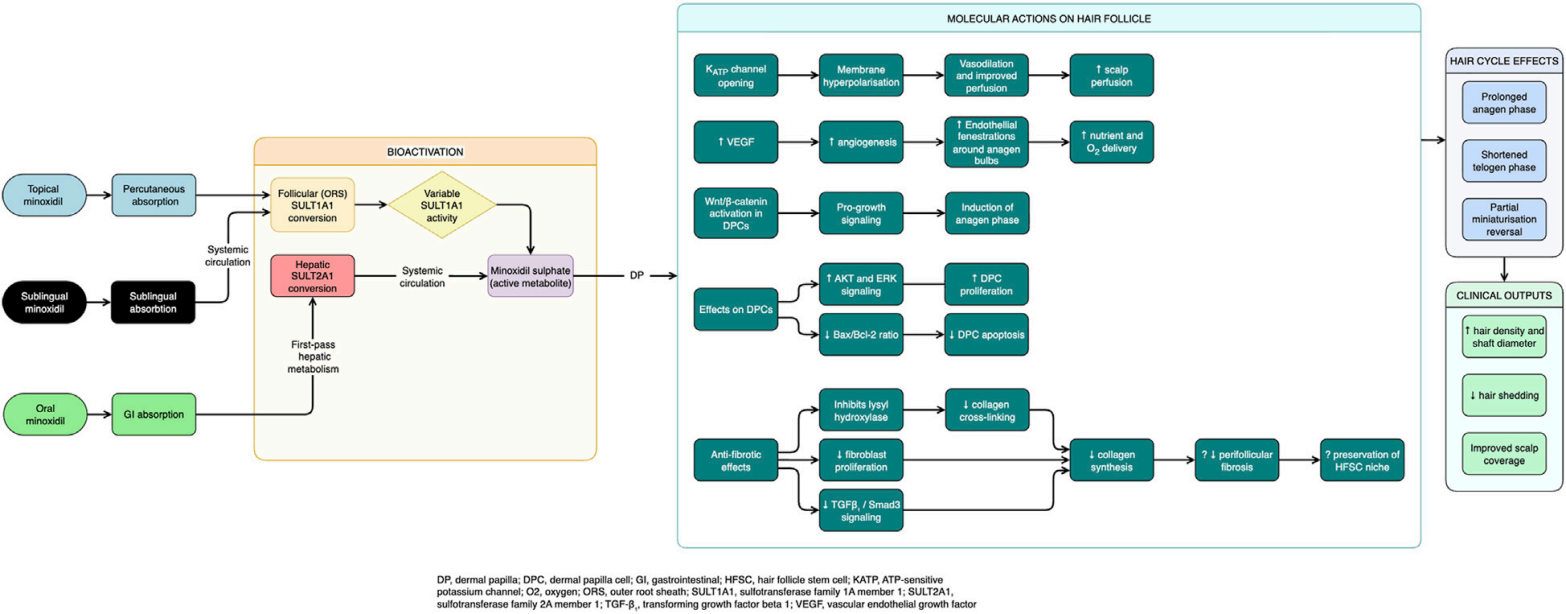
Proposed mechanisms of action on the hair follicle.

Minoxidil is one of the most widely used and clinically validated treatments for AGA. Although it has been used for over 3 decades, its precise mechanism of action in promoting hair growth remains incompletely understood. While minoxidil was initially developed as a potent oral vasodilator for the treatment of hypertension, its hair growth-stimulating properties were discovered serendipitously in the 1970s. Since then, numerous *in vitro*, *in vivo* and *ex vivo* studies have proposed several possible pathways by which minoxidil may enhance HF function. These include potassium channel modulation, angiogenesis, anti-apoptotic effects, prostaglandin modulation and effects on DPC proliferation (Figure 1) (Messenger and Rundegren, 2004).

### 4.1 Activation by sulfotransferase

Minoxidil is a prodrug that requires bioactivation by sulfotransferase (SULT) enzymes, specifically SULT1A1, in the outer root sheath of HFs to produce its active metabolite, minoxidil sulphate (Buhl, 1991; Upton et al., 2015). This enzymatic conversion is critical; without sulphation, minoxidil remains pharmacologically inactive with respect to hair growth. Variability in SULT1A1 activity among individuals is believed to contribute significantly to the variation in clinical response to topical minoxidil (Tosti and Duque-Estrada, 2009).

Roberts et al. (2014) demonstrated that higher follicular SULT1A1 activity is associated with a better response to minoxidil therapy, while non-responders often lack measurable enzymatic activity. These findings have led to the development of diagnostic tests to assess sulfotransferase activity as a predictive marker of treatment efficacy, although these are not yet routine in clinical settings (Roberts et al., 2014).

### 4.2 Pharmacogenomic determinants of minoxidil response

Gene variant analyses (e.g., of *SULT1A1* alleles) have begun to explore whether genotype predicts response to minoxidil therapy. One study identified that *SULT1A1* gene variants were associated with response to oral minoxidil in patients with FPHL (Ramos et al., 2021). Among 10 women who were treated with oral minoxidil 1 mg for 6 months, the average percentage increase in target area hair count was 13.3% in patients with the GG genotype (high SULT1A1 activity) and 6.9% in patients with the GA genotype (low SULT1A1 activity).

However, it should be emphasised that no comprehensive systematic review appears specifically focused on the pharmacogenomics of minoxidil (*SULT1A1* or other genes) in hair loss. Moreover, beyond *SULT1A1*, there are very limited published data linking other genomic loci to minoxidil efficacy or metabolism. Therefore, while *SULT1A1* remains the principal pharmacogenomic biomarker studied to date, future investigations are warranted to integrate emerging genome-wide association study data and broader candidate gene panels (e.g., angiogenesis, potassium channels, etc.) into a precision-treatment paradigm for minoxidil.

### 4.3 Effect on potassium channels

Minoxidil sulphate’s effect on smooth muscle relaxation is related to its ability to open cell surface adenosine triphosphate (ATP)-sensitive potassium (K_ATP_) channels and induce an efflux of potassium, resulting in hyperpolarisation of cell membranes, as demonstrated in *in vitro* smooth muscle and follicular models (Meisheri et al., 1988). Clinically, the observation that chemically unrelated K_ATP_ channel openers such as diazoxide and pinacidil cause hypertrichosis in humans provides indirect *in vivo* support for this mechanism (Koblenzer and Baker, 1968). Furthermore, minoxidil sulphate, as well as other K_ATP_ channel openers such as pinacidil, cromakalin and nicorandil, increase uptake of thymidine and cysteine by mouse vibrissae follicles (Salido et al., 2013).

Cantu syndrome is an autosomal dominant disorder characterised by congenital hypertrichosis, characteristic facial anomalies and cardiomegaly. It is caused by mutations in *ABCC9*, which encodes a regulatory subunit of SUR2, a K_ATP_ channel opener expressed not only in smooth muscle but also in HFs (Ohko et al., 2020). This leads to constitutive activation of the K_ATP_ channel, thus providing a potential mechanistic link to minoxidil’s ability to induce hair growth.

### 4.4 Vascular effects

Minoxidil has been shown to upregulate the expression of vascular endothelial growth factor (VEGF), a potent angiogenic factor (Lachgar et al., 1998). The anagen phase is characterised by perifollicular vascularisation, followed by regression of angiogenic blood vessels during catagen and telogen. Perifollicular angiogenesis is temporally and spatially correlated with upregulation of VEGF expression by outer root sheath keratinocytes of murine HFs (Yano et al., 2001). These findings provide both *in vitro* and animal *in vivo* evidence that minoxidil enhances angiogenesis within the follicular microenvironment. In a rat study, topically applied minoxidil increased capillary fenestration around anagen bulbs without altering total vessel number, further confirming *in vivo* vascular remodelling (Sakita et al., 1999).

### 4.5 Prostaglandin modulation

Another theory suggests that minoxidil may modulate local prostaglandin synthesis. Prostaglandins are lipid mediators known to influence HF cycling. Specifically, prostaglandin E2 (PGE2) and prostaglandin F2α (PGF2α) promote hair growth whereas prostaglandin D2 (PGD2) is associated with hair growth inhibition and miniaturisation (Johnstone and Albert, 2002). Prostaglandins are produced from arachidonic acid by isoforms of prostaglandin endoperoxide-H synthase (PGHS). Minoxidil is a potent activator of the enzyme PGHS-1, which catalyses the conversion of arachidonic acid to prostaglandin endoperoxide, which is itself a precursor to a variety of prostaglandins (Michelet et al., 1997). To date, this mechanism remains primarily supported by *in vitro* biochemical studies, with no direct *in vivo* validation.

### 4.6 Dermal papilla cell proliferation and apoptosis

It is well known that the number of DPCs in the HFs correlates with hair size and shape (Elliott et al., 1999). Evidence derived from *in vitro* studies of cultured human DPCs demonstrates that minoxidil promotes DPC proliferation and survival by upregulating AKT and ERK signalling. In addition, it prevents apoptosis of DPCs by increasing the Bcl-2/Bax ratio (Han et al., 2004). In addition, VEGF upregulation may further stimulate DPC proliferation (Lachgar et al., 1998). While consistent with clinical outcomes, the molecular confirmation of these signalling effects in human *in vivo* scalp tissue has not yet been demonstrated.

### 4.7 Wnt/β-catenin signalling pathway

The Wnt/β-catenin signalling pathway plays a critical role in the development, initiation and growth of HFs (Kishimoto et al., 2000). β-catenin activity in DPCs is required for regeneration of HFs and maintenance of the anagen phase (Enshell-Seijffers et al., 2010). Topical minoxidil induces nuclear accumulation of β-catenin in DPCs in murine *in vivo* models, implicating this pathway in anagen maintenance.

### 4.8 Anti-fibrotic effects

Histopathological studies have demonstrated that AGA is frequently accompanied by perifollicular fibrosis, characterised by concentric collagen deposition around miniaturising follicles (Trüeb, 2010; Trueb, 2002; Heymann, 2022; Whiting, 1993). While AGA is classified as a non-scarring alopecia, the presence of perifollicular fibrosis suggests a connective tissue remodelling component that contributes to its progressive nature.

Evidence that minoxidil directly modulates fibrotic remodelling in human scalp is currently indirect. In *in vitro* fibroblast assays and non-cutaneous *in vivo* models such as bleomycin-induced pulmonary fibrosis in mice, minoxidil has been shown to inhibit lysyl hydroxylase activity and fibroblast proliferation, thereby decreasing collagen synthesis (Knitlova et al., 2021; Shao et al., 2018; Parish et al., 1995). These findings suggest a theoretical anti-fibrotic potential, but its clinical relevance to human AGA pathology remains unproven and should be interpreted cautiously. Future studies using scalp biopsy or molecular imaging approaches are warranted to clarify whether minoxidil exerts measurable anti-fibrotic effects in AGA.

Collectively, the above mechanisms span both *in vitro* and *in vivo* evidence, with the most robust experimental support for VEGF-mediated angiogenesis and β-catenin signalling, while other proposed mechanisms (prostaglandin modulation, anti-fibrotic activity) remain hypothetical pending further human *in vivo* validation.

## 5 Topical minoxidil

**FIGURE 2:**
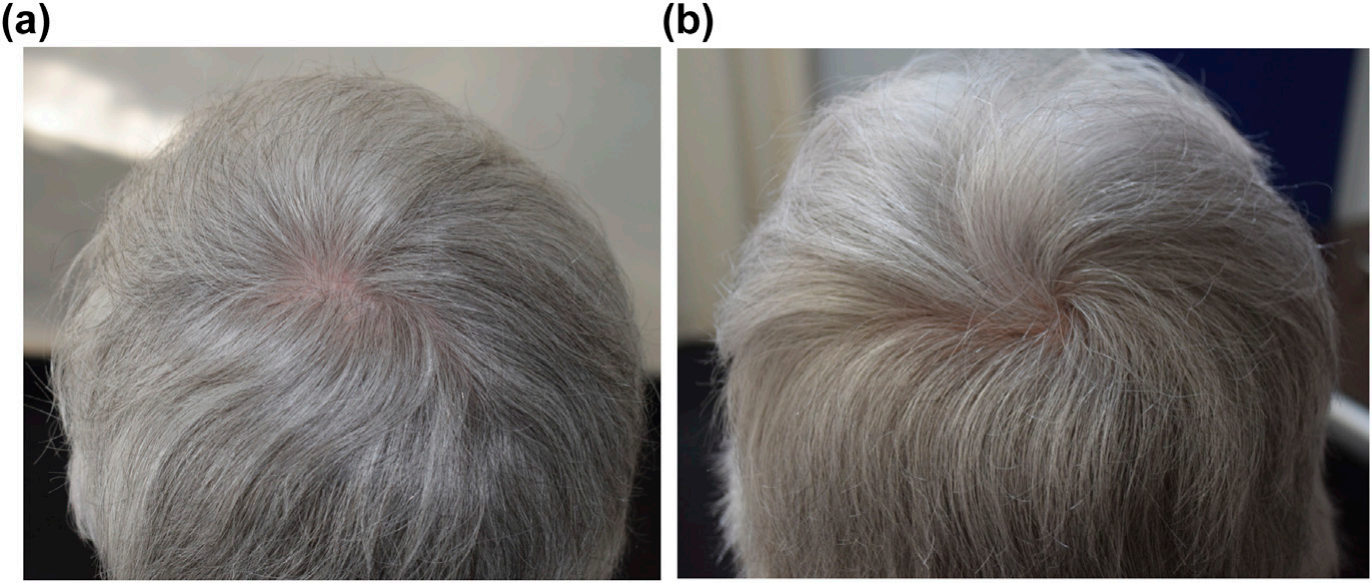
Vertex scalp photographs of a 65-year-old woman with female pattern hair loss **(a)** before and **(b)** after 15 months of treatment with 5% minoxidil foam.

Topical minoxidil is the most extensively studied and widely used therapy for AGA in both men and women (Figure 2). Since its initial FDA approval, it has remained the mainstay of treatment due to its established efficacy, favourable safety profile and accessibility.

The solution formulation contains ethanol and propylene glycol, which are used as vehicles to enhance the solubility of minoxidil (Tata et al., 1994). On the other hand, ingredients in the foam formulation include cetyl alcohol, stearyl alcohol and butylated hydroxytoluene (Gogtay and Panda, 2009).

### 5.1 Efficacy in male pattern hair loss

The efficacy of topical minoxidil in MPHL has been consistently demonstrated in RCTs, meta-analyses and real-world observational studies. The most robust data come from a double-blind RCT by Olsen et al. (2002), in which 393 men with vertex hair loss were randomised to receive 5% minoxidil solution, 2% minoxidil solution or a vehicle solution twice daily for 48 weeks. At the end of the study, the 5% minoxidil group showed a mean increase of 18.6 non-vellus hairs/cm^2^ from baseline, compared to 12.7 hairs/cm^2^ in the 2% minoxidil group and 3.9 hairs/cm^2^ in the placebo group. Global photographic assessments favoured the 5% formulation, with 60% of participants rated as “improved” versus 23% in the placebo arm.

The 5% foam formulation has demonstrated similar efficacy with improved tolerability. In a 16-week multicentre RCT involving 352 men with AGA treated with 5% minoxidil foam or placebo, hair counts in the target area increased by 13.4% in the treatment group versus 3.4% in the placebo group (p < 0.001). Investigator and subject assessments both reflected significant improvements in hair coverage and cosmetic acceptability in the minoxidil foam arm (Olsen et al., 2007). In an open-label clinical trial of men with MPHL treated with 5% minoxidil foam or placebo, target area non-vellus hair counts and cumulative hair width in the frontotemporal and vertex areas of the scalp showed a statistically significant increase from baseline to weeks 52 and 76, returning to values comparable to baseline at week 104 (Kanti et al., 2016).

A 2022 network meta-analysis of 23 studies evaluating pharmacological treatments for MPHL concluded that 5% minoxidil solution had superior efficacy to 2% minoxidil solution (Gupta et al., 2022b). Notably, efficacy was more pronounced when treatment was maintained for at least 24 weeks, reflecting the length of the hair cycle.

### 5.2 Efficacy in female pattern hair loss

Favourable outcomes have also been demonstrated in women with FPHL treated with topical minoxidil. In a pivotal RCT of 381 women with FPHL treated with topical minoxidil 5%, topical minoxidil 2% or placebo, improvements in hair counts and investigator assessments of hair growth or scalp coverage were greatest in the topical minoxidil 5% group, followed by the topical minoxidil 2% group. Changes in non-vellus hair counts from baseline were 24.5, 20.7 and 9.4 in a 1 cm^2^ target area respectively for the topical minoxidil 5%, topical minoxidil 2% and placebo groups (p < 0.001) (Lucky et al., 2004).

Blume-Peytavi et al. (2011) conducted a 24-week single-blind RCT comparing once-daily 5% foam with twice-daily 2% solution in 113 women with AGA. The 5% foam group achieved a greater mean increase in non-vellus hair count (+31.9/cm^2^ vs. +28.4/cm^2^) from baseline although the difference was not statistically significant. In another RCT which enrolled 404 women with FPHL, 5% topical minoxidil foam resulted in regrowth of 10.9 hairs/cm^2^ and 9.1 hairs/cm^2^ more than a vehicle foam after 12 and 24 weeks respectively (Bergfeld et al., 2016). Furthermore, the total unit area density (sum of hair diameters/cm^2^) increased by 658 μm/cm^2^ and 644 μm/cm^2^ more with 5% minoxidil foam than with the vehicle foam after 12 and 24 weeks respectively.

In a network meta-analysis which used data from 13 trials to determine the comparative efficacy of minoxidil and 5α-reductase inhibitors in the treatment of FPHL, 5% minoxidil foam was found to be superior to 2% and 5% minoxidil solutions over a 24-week period (Gupta et al., 2022b).

### 5.3 Predictors of response

Despite the general efficacy of topical minoxidil, approximately 60% of patients are considered “non-responders.” This variability is attributed in part to differences in follicular sulfotransferase activity. Goren et al. (2014) demonstrated that patients with high follicular sulfotransferase activity were significantly more likely to respond to treatment.

Approximately 1.4% of minoxidil applied topically on a healthy scalp is absorbed, with differences in absorption linked to its concentration, frequency of application and skin integrity (Rossi et al., 2012). Adjunctive procedures such as microneedling and laser-assisted drug delivery may enhance efficacy although evidence for this is still lacking.

Duration and severity of hair loss, and compliance also influence treatment outcomes. Longstanding AGA may result in follicular dropout and, consequently, irreversible hair loss (Sperling, 2001). Therefore, better responses to topical minoxidil treatment are typically observed in patients with early-stage disease.

### 5.4 Safety and tolerability

Topical minoxidil is generally well tolerated, with a favourable safety profile that supports its use across a wide population. The most common side effects include pruritus and increased hair shedding during the initial phase of treatment (Hussein et al., 2024). Scalp irritation is more frequent with the alcohol-based solution due to its propylene glycol content. The 5% foam, which lacks propylene glycol, has been associated with significantly reduced irritation and higher patient adherence (Blume-Peytavi et al., 2011).

The risk of hypertrichosis is influenced by the concentration of topical minoxidil, with individuals treated with 5% minoxidil solution more likely to develop unwanted hair growth. After discontinuation of minoxidil treatment, spontaneous resolution of hypertrichosis typically begins on the face and arms within 1–3 months, followed by the legs within 4–5 months (Dawber and Rundegren, 2003).

Systemic adverse effects such as hypotension or tachycardia are exceedingly rare in healthy individuals treated with topical minoxidil.

## 6 Oral minoxidil

**FIGURE 3:**
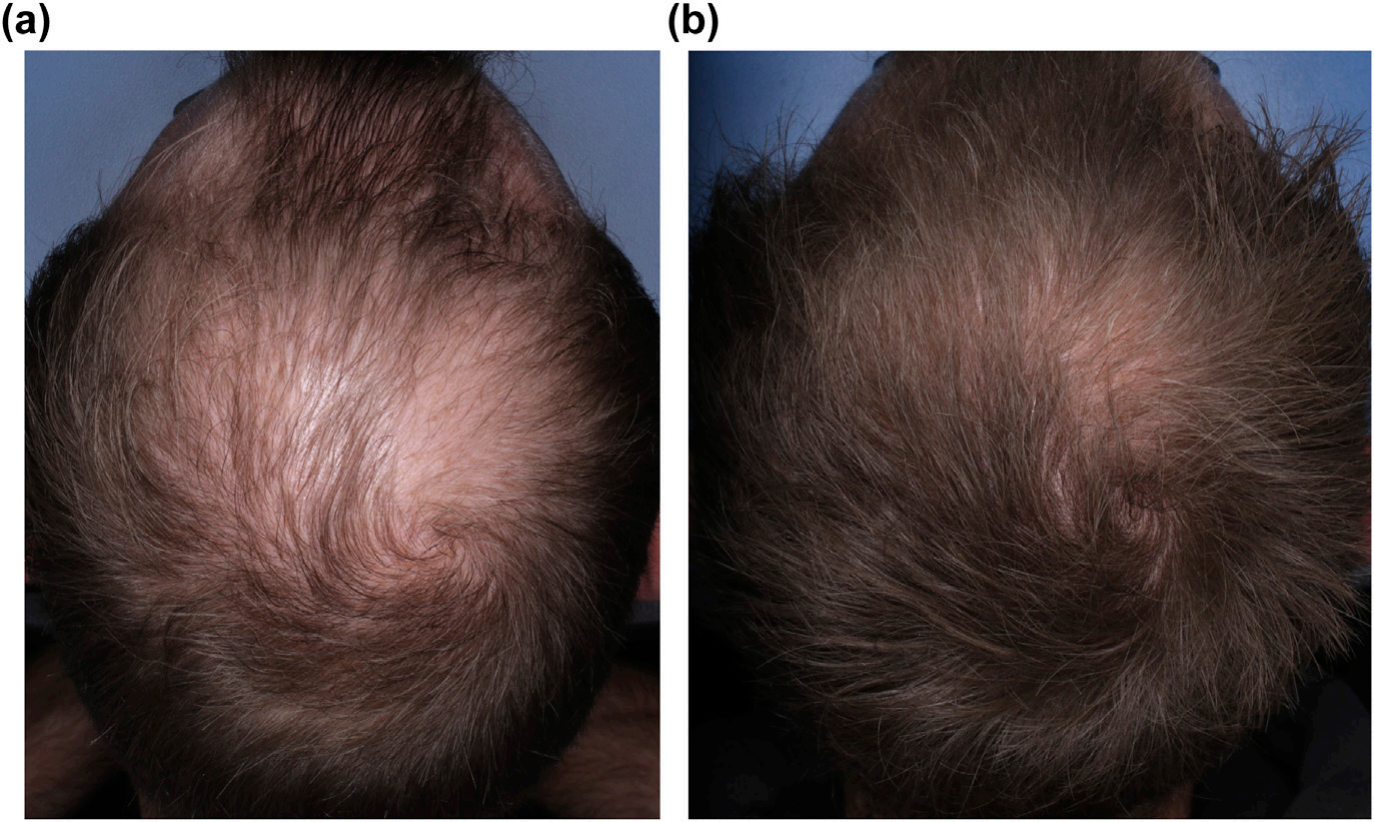
Vertex scalp photographs of a 30-year-old man with male pattern hair loss **(a)** before and **(b)** after 4 years of treatment with dutasteride 0.5 mg and oral minoxidil 7.5 mg. The dose of oral minoxidil was progressively uptitrated from an initial dose of 1 mg. Adjuvant therapies with platelet-rich plasma (six sessions) and intradermal injections of dutasteride (three sessions) were administered for 2 years.

Oral minoxidil, originally approved for the treatment of refractory hypertension, has gained increasing attention as an effective off-label treatment for AGA (Figure 3). LDOM, typically administered in doses ranging from 0.25 to 5 mg, is emerging as a viable alternative for patients who fail to respond to or experience side effects with topical therapy, or seek a more convenient systemic option.

Although oral minoxidil is not formally approved for AGA, its use is supported by a growing body of evidence from prospective studies, retrospective case series and a small number of RCTs. These studies suggest that LDOM offers clinically significant improvements in hair density, patient satisfaction and overall cosmetic outcomes, and is well tolerated.

### 6.1 Rationale for oral administration

Low SULT1A1 activity occurs in approximately 60% of the population and predicts a poor response to topical minoxidil (Ramos et al., 2021; Goren et al., 2014; Ramos et al., 2020a). Poor responders to topical minoxidil can respond to oral minoxidil as the latter is converted into minoxidil sulphate in the liver by the SULT2A1 isoenzyme. Consequently, systemic delivery ensures more consistent availability of minoxidil sulphate in the HF.

The minimum plasma threshold of minoxidil required to induce hair growth is 0.7 ng/mL, lower than the threshold required for haemodynamic effects (18 ng/mL). Therefore, lower doses of oral minoxidil than those used to treat hypertension can induce hair growth.

### 6.2 Efficacy in male pattern hair loss

A retrospective analysis of 41 men with MPHL treated with oral minoxidil 2.5 mg or 5 mg demonstrated clinical improvement in 90% of cases (Jimenez-Cauhe et al., 2019). The efficacy of oral minoxidil in the treatment of MPHL and FPHL has been evaluated in a small number of clinical trials. In a double-blind placebo-controlled RCT which enrolled men with MPHL for 24 weeks, the difference in terminal and total hair counts between patients treated with oral minoxidil 5 mg and twice-daily topical minoxidil 5% was 3.1/cm^2^ and 2.6/cm^2^ on the frontal scalp respectively, and 23.4/cm^2^ and 5.5/cm^2^ on the vertex scalp respectively (Penha et al., 2024). However, the above differences between the two groups were not statistically significant. In another RCT of 65 patients with AGA who received either topical minoxidil 5% or oral minoxidil 1 mg for 6 months, both groups demonstrated significant improvements in hair diameter (Asilian et al., 2024). Photographic assessment demonstrated a significant improvement in hair density in the topical minoxidil group but not in the oral minoxidil group, but the difference between the two groups was not statistically significant.

A Bayesian network meta-analysis to determine the comparative effect of monotherapies for MPHL found dutasteride to be the top-most ranked regimen for both 6-month change in total (surface under the cumulative ranking curve, SUCRA = 86.9%) and terminal (SUCRA = 98.2%) hair densities (Gupta et al., 2024). Although “oral minoxidil 5 mg once daily for 24 weeks” was ranked lower than “dutasteride 0.5 mg once daily for 24 weeks”, the difference in these two regimens’ effect was not statistically significant for change in total and terminal hair density at 6 months.

### 6.3 Efficacy in female pattern hair loss

The use of LDOM to treat AGA was first reported in 2018 in 100 women (Sinclair, 2018). After 12 months of treatment with minoxidil 0.25 mg and spironolactone 25 mg, a mean reduction in hair loss severity and hair shedding scores of 1.3 and 2.6 respectively were observed.

Ramos et al. (2020b) compared oral minoxidil 1 mg and once-daily topical minoxidil 5% in the treatment of women with FPHL. After 24 weeks of treatment, the total hair density increased by 12% in women taking oral minoxidil and 7.2% in women applying topical minoxidil, but the difference between the two groups was not statistically significant. However, the reduction in hair shedding was superior in the oral minoxidil group. Another RCT compared a lower dose of oral minoxidil (0.25 mg) with topical minoxidil 2% in the treatment of FPHL in 72 women. In the oral minoxidil group, the mean hair fibre diameter and hair density increased by 4 μm and 13/cm^2^ respectively after 9 months. In the topical minoxidil group, the mean hair fibre diameter and hair density increased by 3 μm and 6/cm^2^ respectively in the same time period. The differences between the oral minoxidil and topical minoxidil groups were not statistically significant (Vahabi-Amlashi et al., 2021).

### 6.4 Safety and tolerability

While oral minoxidil offers substantial efficacy, it carries a risk of systemic side effects due to its vasodilatory action. Nonetheless, adverse events are dose-dependent and infrequent when using low-dose regimens.

In a multicentre study of 1404 men and women with various hair loss disorders, LDOM was well tolerated (Vano-Galvan et al., 2021). The most frequent adverse event was hypertrichosis in 15.1% of patients. Cardiovascular adverse effects were rare and included fluid retention (1.3%), lightheadedness (1.7%) and tachycardia (0.9%). The above cardiovascular adverse events were time-dependent, and all resolved with dose reduction or treatment discontinuation. Only 1.7% of patients in this study required treatment cessation due to adverse effects. Many of the above side effects are dose-dependent, with hypertrichosis affecting 6.7% of patients treated with oral minoxidil 0.25 mg and 56.1% of patients treated with oral minoxidil 5 mg (Jimenez-Cauhe et al., 2020).

It is encouraging that LDOM also has a favourable safety profile in patients with pre-existing cardiovascular diseases such as hypertension or arrhythmia. In a retrospective study of 264 patients with pre-existing hypertension or arrhythmia treated with LDOM for hair loss, 9% experienced a systemic adverse effect (Jimenez-Cauhe et al., 2024). The most common reason for LDOM dose adjustment or withdrawal was postural hypotension. Patients who were on three or more antihypertensive drugs had a higher risk of developing more than one adverse effect. Additionally, among patients with hypertension who experienced cardiovascular adverse effects, dose adjustment or discontinuation of antihypertensive drugs were necessary in 2 cases. No patients with comorbid arrhythmias reported palpitations or other cardiovascular adverse effects. Importantly, no life-threatening adverse effects were reported. In a prospective study of hypertensive patients using LDOM for hair loss, no significant changes in blood pressure or heart rate were observed (Muller Ramos et al., 2024).

Rare but serious adverse effects associated with higher doses of oral minoxidil (>10 mg) e.g., pericardial effusion are less likely with doses ≤5 mg (Nakarmi et al., 2025). Routine blood pressure monitoring is not strictly necessary for healthy patients but should be considered for those with a history of cardiovascular disease, hypotension or concomitant antihypertensive drugs.

Limited data suggest that oral minoxidil is reasonably safe in patients with a contact allergy to topical minoxidil. Nine patients with positive patch tests to 5% minoxidil solution and 5% minoxidil foam but not propylene glycol were treated with oral minoxidil 0.25 mg twice daily for an average duration of 17 months without developing any side effects (Therianou et al., 2022).

## 7 Sublingual minoxidil

**FIGURE 4:**
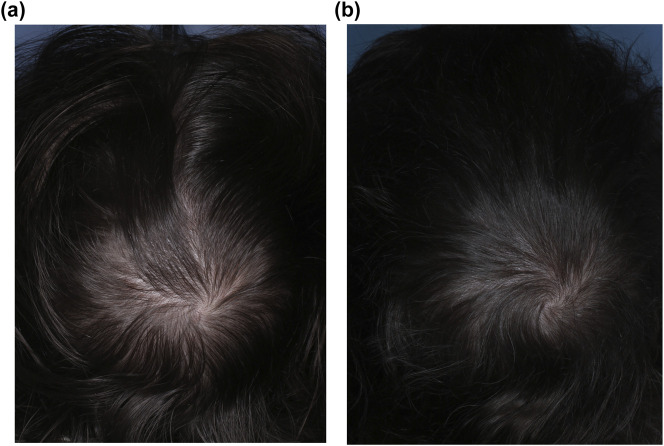
Vertex scalp photographs of a 39-year-old woman with female pattern hair loss **(a)** before and **(b)** after 63 months of treatment with bicalutamide 10 mg and sublingual minoxidil 3.6 mg. The dose of sublingual minoxidil was progressively uptitrated from an initial dose of 0.45 mg.

Sublingual minoxidil is a novel formulation under investigation for the treatment of AGA (Figure 4). Although still in early stages of clinical evaluation, sublingual minoxidil appears promising, with early studies reporting meaningful hair regrowth and a favourable safety profile with low doses.

### 7.1 Rationale for sublingual administration

As with follicular SULT1A1, there is considerable interindividual heterogeneity in levels of hepatic SULT2A1, resulting in a variable response of patients with AGA to oral minoxidil. Administration of oral minoxidil results in higher levels of minoxidil sulphate in the circulation, thereby increasing the risk of haemodynamic side effects such as orthostatic hypotension and reflex tachycardia. Furthermore, minoxidil sulphate diffuses poorly from the circulation to the skin and, consequently, the HF due to its high molecular weight. On the other hand, sublingual minoxidil bypasses first-pass hepatic metabolism and is absorbed directly into the bloodstream via the oral mucosa (Sinclair et al., 2020). It eventually reaches the HF where it is converted to minoxidil sulphate by SULT1A1, thereby optimising the bioavailability of minoxidil sulphate at the level of the anagen hair bulb. In patients with low levels of follicular SULT1A1, higher doses of sublingual minoxidil can be used to achieve the desired therapeutic effect.

### 7.2 Efficacy in male and female pattern hair loss

To date, only 4 studies have evaluated the efficacy of sublingual minoxidil in the treatment of AGA. A retrospective study of 33 men and 31 women with AGA treated with sublingual minoxidil at a dose of 0.45 mg or 0.9 mg demonstrated a mean reduction in Sinclair grade of 0.33 at 3 months, 0.53 at 6 months and 1.07 at 12 months (Sinclair et al., 2020). The same group performed a double-blind clinical trial in which 40 men and women with AGA were randomised to sublingual minoxidil 0.45 mg or placebo (Bokhari et al., 2022). Twelve participants rolled into a 24-week open-label extension study and received sublingual minoxidil 1.35 or 4.05 mg. Phototrichograms demonstrated a mean increase in terminal hair counts of 4/cm^2^, 10/cm^2^ and 38/cm^2^ on the frontal scalp in patients treated with sublingual minoxidil 0.45 mg, 1.35 mg and 4.05 mg respectively. On the vertex scalp, a mean increase in terminal hair counts of 9/cm^2^, 26/cm^2^ and 88/cm^2^ was observed in patients treated with sublingual minoxidil 0.45 mg, 1.35 mg and 4.05 mg respectively.

In an open-label study comparing sublingual minoxidil with placebo in men with MPHL, authors measured changes in hair shaft diameter rather than hair counts (Sinclair et al., 2025). After 24 weeks, fibre diameter decreased by 2 μm in men receiving placebo. Among men receiving sublingual 0.45 mg, 1.35 mg and 4.05 mg, the mean change in fibre diameter was −2 μm, 3 μm and 6 μm respectively. A dose-dependent increase in mean fibre diameter was seen in men who received doses of 1.35 mg and 4.05 mg, with the increase being greatest in terminal and intermediate hairs compared with vellus hairs.

The question of whether sublingual administration offers any advantages over oral minoxidil was addressed in a recent RCT which compared sublingual minoxidil 5 mg with oral minoxidil 5 mg in 110 men with MPHL (Sanabria et al., 2025). The total and non-vellus hair density increased by 24.5/cm^2^ and 7.8/cm^2^ respectively in the sublingual group, and 21.8/cm^2^ and 7.4/cm^2^ respectively in the oral group. However, these differences were not statistically significant.

In a retrospective study evaluating the long-term efficacy of systemic minoxidil, 51 women with FPHL were treated with oral or sublingual minoxidil for a minimum of 3 years (Kushnir-Grinbaum et al., 2025). A 1-point reduction in hair loss severity grade was observed in 47.1% and 64.7% of cases after 3 and 5 years respectively, suggesting a cumulative effect of systemic minoxidil on hair growth over a period of 5 years.

### 7.3 Safety and tolerability

The safety profile of sublingual minoxidil has so far mirrored that of LDOM, with a low incidence of systemic side effects. In a retrospective study of 64 patients treated with sublingual minoxidil, adverse effects were mild and included hypertrichosis (12.5%), postural hypotension (7.8%) and peripheral oedema (3.1%) (Sinclair et al., 2020). No serious side effects were reported. No significant changes in systolic and diastolic blood pressure were observed at 6 and 12 months. Similarly, in the clinical trial conducted by Bokhari et al. (2022), no significant changes in blood pressure were detected in patients treated with all 3 doses of sublingual minoxidil.

In the RCT which compared sublingual minoxidil 5 mg with oral minoxidil 5 mg in men with MPHL, palpitations occurred in 0% in the sublingual group versus 9.3% in the oral group (p = 0.048) (Sanabria et al., 2025). However, there were no statistically significant differences in frequency of other side effects, including hypertrichosis, lightheadedness and oedema, between the two groups.

Adverse effects of both oral and sublingual minoxidil are summarised in Table 1. Across the four pooled cohorts, hypertrichosis was consistently the most frequent adverse effect, with a combined incidence of approximately 15% (Vano-Galvan et al., 2021; Sanabria et al., 2025; Sinclair et al., 2020; Sanabria et al., 2021). Rates were higher in women than in men — 20% versus 6% for oral administration and 12.5% versus 0% for sublingual administration (Vano-Galvan et al., 2021; Sinclair et al., 2020). A separate cohort of women treated with oral minoxidil reported a substantially higher frequency of adverse effects (65%), suggesting population-specific variability (Sanabria et al., 2021). Cardiovascular or fluid-related events were uncommon overall: dizziness, tachycardia and lower limb oedema occurred in 1%-9%, 1%-9% and 0-3% of patients respectively depending on the route of administration and dose of minoxidil, and study population. These findings derive from a pooled descriptive synthesis of 4 independent studies encompassing oral and sublingual minoxidil with varying doses and treatment durations. Given the heterogeneity in methodology, sample size and sex-specific reporting across studies, pooled estimates should be interpreted with caution.

**TABLE 1:** Adverse effects of oral and sublingual minoxidil.

Study reference	Sex	Sample size	Adverse effect	Route	Incidence	Required dose adjustment	Required discontinuation
Vano-Galvan et al. (2021)	Female	1612	Hypertrichosis	Oral	20%	5%	1%
Vano-Galvan et al. (2021)	Male	857	Hypertrichosis	Oral	6%	1%	0
Sinclair et al. (2020)	Female	31	Hypertrichosis	Sublingual	12.5%	n/a	n/a
Sinclair et al. (2020)	Male	33	Hypertrichosis	Sublingual	0	n/a	n/a
Sanabria et al. (2021)	Female	215	Hypertrichosis	Oral	65%	n/a	n/a
Sanabria et al. (2021)	Male	220	Hypertrichosis	Oral	49%	n/a	n/a
Vano-Galvan et al. (2021)	Female	1612	Dizziness	Oral	2%	n/a	n/a
Vano-Galvan et al. (2021)	Male	857	Dizziness	Oral	1%	n/a	10% (n = 10)
Sanabria et al. (2025)	Male	42	Dizziness	Oral	5%	n/a	n/a
Sanabria et al. (2025)	Male	43	Dizziness	Sublingual	2%	n/a	n/a
Sinclair et al. (2020)	Female	31	Dizziness	Sublingual	6%	n/a	n/a
Sinclair et al. (2020)	Male	33	Dizziness	Sublingual	9%	n/a	n/a
Sanabria et al. (2021)	Female	215	Dizziness	Oral	7%	n/a	n/a
Sanabria et al. (2021)	Male	220	Dizziness	Oral	7%	n/a	n/a
Vano-Galvan et al. (2021)	Female	1612	Lower limb oedema	Oral	1%	n/a	33% (n = 18)
Vano-Galvan et al. (2021)	Male	857	Lower limb oedema	Oral	2%	n/a	14% (n = 14)
Sanabria et al. (2025)	Male	42	Lower limb oedema	Oral	2%	n/a	n/a
Sanabria et al. (2025)	Male	43	Lower limb oedema	Sublingual	2%	n/a	n/a
Sinclair et al. (2020)	Female	31	Lower limb oedema	Sublingual	3%	n/a	n/a
Sinclair et al. (2020)	Male	33	Lower limb oedema	Sublingual	0	n/a	n/a
Sanabria et al. (2021)	Female	215	Lower limb oedema	Oral	9%	n/a	n/a
Sanabria et al. (2021)	Male	220	Lower limb oedema	Oral	3%	n/a	n/a
Vano-Galvan et al. (2021)	Male	857	Tachycardia	Oral	1%	n/a	n/a
Sanabria et al. (2025)	Male	42	Tachycardia	Oral	9%	n/a	n/a
Sanabria et al. (2025)	Male	43	Tachycardia	Sublingual	0	n/a	n/a
Sanabria et al., (2021)	Female	215	Tachycardia	Oral	4%	n/a	n/a
Sanabria et al. (2021)	Male	220	Tachycardia	Oral	4%	n/a	n/a
Vano-Galvan et al. (2021)	Male	857	Headache	Oral	0.1%	n/a	0
Sanabria et al. (2025)	Male	42	Headache	Oral	12%	n/a	n/a
Sanabria et al. (2025)	Male	43	Headache	Sublingual	4%	n/a	n/a
Sanabria et al. (2021)	Female	215	Headache	Oral	10%	n/a	n/a
Sanabria et al. (2021)	Male	220	Headache	Oral	8%	n/a	n/a
Vano-Galvan et al. (2021)	Male	857	Facial or periorbital oedema	Oral	0.3%	n/a	0
Sanabria et al. (2025)	Male	42	Facial or periorbital oedema	Oral	2%	n/a	n/a
Sanabria et al. (2025)	Male	43	Facial or periorbital oedema	Sublingual	2%	n/a	n/a
Sanabria et al. (2021)	Female	215	Facial or periorbital oedema	Oral	1%	n/a	n/a
Sanabria et al. (2021)	Male	220	Facial or periorbital oedema	Oral	1%	n/a	n/a
Vano-Galvan et al. (2021)	Male	857	Insomnia	Oral	0.1%	n/a	0
Sanabria et al. (2025)	Male	42	Insomnia	Oral	7%	n/a	n/a
Sanabria et al. (2025)	Male	43	Insomnia	Sublingual	8%	n/a	n/a
Sanabria et al. (2021)	Female	215	Insomnia	Oral	7%	n/a	n/a
Sanabria et al. (2021)	Male	220	Insomnia	Oral	7%	n/a	n/a
Sanabria et al. (2021)	Female	215	Increased appetite	Oral	2%	n/a	n/a
Sanabria et al. (2021)	Male	220	Increased appetite	Oral	1%	n/a	n/a
Sanabria et al. (2021)	Female	215	Indigestion	Oral	1%	n/a	n/a
Sanabria et al. (2021)	Male	220	Indigestion	Oral	1%	n/a	n/a
Sanabria et al. (2021)	Female	215	Syncope	Oral	0	n/a	n/a
Sanabria et al. (2021)	Male	220	Syncope	Oral	1%	n/a	n/a
Sanabria et al. (2021)	Female	215	Dry mouth	Oral	0	n/a	n/a
Sanabria et al. (2021)	Male	220	Dry mouth	Oral	1%	n/a	n/a
Sanabria et al. (2021)	Female	215	Hair shedding	Oral	44%	n/a	n/a
Sanabria et al. (2021)	Male	220	Hair shedding	Oral	21%	n/a	n/a
Sanabria et al. (2021)	Female	215	Nightmares	Oral	2%	n/a	n/a
Sanabria et al. (2021)	Male	220	Nightmares	Oral	2%	n/a	n/a

## 8 Overdose of minoxidil

### 8.1 Overdose of topical minoxidil

Most intoxications follow ingestion of the 5% solution, although excessive topical application can also cause systemic effects, e.g., prolonged dizziness (Ponomareva et al., 2024). In 3 reports, ingestion of an entire 60 mL bottle (equivalent to 3000 mg) resulted in severe circulatory shock (Farrell and Epstein, 1999; Tripathee et al., 2024; Panchal et al., 2014), with systolic blood pressure even dropping to 40 mmHg in 1 case (Farrell and Epstein, 1999). Even smaller overdoses (10–30 mL) have been associated with tachycardia and hypotension (Chakar et al., 2023; Shashikala et al., 2016; Mazhar et al., 2023). All the above cases necessitated treatment with vasoactive agents such as dopamine, norepinephrine and phenylephrine. A single case of myocardial infarction followed ingestion of 60 mL of 2% minoxidil solution (MacMillan et al., 1993), while 2 cases of unstable angina have been reported after ingestion of 5 mL and 60 mL of 5% minoxidil solution (Panchal et al., 2014; Gheshlaghi et al., 2018). The above cases may have been the result of an increase in myocardial oxygen demand, and coronary hypoperfusion secondary to tachycardia and hypotension. Paediatric exposure to only 3–5 mL of 5% minoxidil solution (equivalent to 150–250 mg) caused drowsiness, tachycardia, hypotension and electrocardiogram (ECG) changes (T wave inversion) but resolved within 20 h with supportive care and inotropic support (Chakar et al., 2023).

### 8.2 Overdose of oral minoxidil

Overdoses of oral minoxidil are less common but produce similar toxicity. A 61-year-old man developed hypotension, prominent oedema, facial flushing and rapid weight gain of nearly 12 kg after ingesting 350 mg (35 × 10 mg tablets) of oral minoxidil (Kakurai and Moriyama, 2025). Compounding errors can result in patients receiving higher doses of minoxidil than those prescribed by their dermatologists. In 1 series, patients developed severe adverse effects including tachycardia, hypotension, syncope and generalised oedema after taking up to 1000 mg of minoxidil, with 2 patients suffering a myocardial infarction and a stroke (Moreno-Arrones et al., 2022). A healthy 43-year-old woman suffered an acute myocardial infarction after taking capsules containing 300–400 mg of minoxidil, although the dose prescribed by her dermatologist was 0.5 mg (Óscar Fabregat Andrés, 2021). These cases underscore the importance of accurate pharmacy preparation.

Hypotension associated with overdoses from both topical and oral minoxidil is often refractory to fluids alone; α1 agonists (e.g., phenylephrine, norepinephrine or midodrine) may be required. Fluid overload, including pleural effusions, may complicate large ingestions and often responds to diuretic treatment. No fatalities were reported among the cases summarised.

## 9 Future prospects

AGA is the most common form of non-scarring alopecia worldwide, mediated by genetic predisposition and excessive follicular sensitivity to androgens. It is a chronic condition characterised by progressive loss of terminal scalp hair. Although highly prevalent and not life-threatening, it is associated with a substantial psychosocial burden. Significant advances have been made in understanding the pathophysiology of AGA, yet only 2 drugs remain FDA-approved: finasteride and topical minoxidil. Minoxidil is a cornerstone of management and is considered a first-line therapy, with topical formulations approved for both men and women. RCTs have consistently demonstrated its efficacy. However, adherence is often limited by local adverse effects, cosmetic acceptability and the fact that approximately 60% of users do not achieve visible improvement. Evidence indicates that better responses are typically observed in patients with early-stage disease. These limitations have stimulated growing interest in systemic alternatives.

LDOM has emerged as a practical option for patients who fail to respond to or tolerate topical therapy. Clinical evidence from observational studies and RCTs has consistently shown improvements in hair density and hair shaft diameter, with hypertrichosis the most common adverse effect. Cardiovascular events such as oedema, tachycardia or postural hypotension are uncommon at doses ≤5 mg and are usually manageable with dose adjustment or discontinuation. Increasingly, LDOM is being integrated into routine practice, although its use remains off-label and requires careful patient selection and monitoring.

Sublingual minoxidil represents the newest development. By bypassing hepatic first-pass metabolism, sublingual minoxidil offers the possibility to increase follicular delivery of the parent drug while reducing systemic exposure to minoxidil sulphate, the active metabolite. Early clinical studies suggest comparable efficacy to oral administration, with a possible reduction in the risk of cardiovascular side effects. While data are still limited to small trials and case series, results are encouraging and may justify broader investigation in larger, long-term studies.

Differences in treatment outcomes among the different formulations of minoxidil are shown in Table 2. It is important to note the significant methodological heterogeneity across studies, including variations in design, study population, dosing regimen, treatment duration and outcome assessment, making direct comparisons difficult.

**TABLE 2:** Summary of clinical studies assessing changes in hair density or hair fibre diameter after treatment with topical, oral and sublingual minoxidil.

Study reference	Study groups and interventions	Population	Study design	Sample size	Duration	Total hair density (baseline → final), hairs/cm^2^	Mean Δ hair density, hairs/cm^2^	Mean Δ hair fibre diameter
Ramos et al. (2020b)	Oral minoxidil 1 mg vs. minoxidil 5% solution	Women with FPHL	Open-label RCT	52	24 weeks	OM: 164.6 (SD 48.1) → 184.7 (SD 57.1) TM: 163.2 (SD 46.0) → 176.3 (SD 61.5)	OM: +20.1 (+12%) TM: +13.1 (+7.2%)	Not reported
Vahabi-Amlashi et al. (2021)	Oral minoxidil 0.25 mg + topical placebo vs. topical minoxidil 2% + oral placebo	Women with FPHL	Triple-blind RCT	72	9 months	OM: 102.0 (SD 79.2) → 109.8 (SD 79.2) TM: 107.4 (SD 21.0) → 115.3 (SD 133.2)	OM: +7.8 (+7.6%) TM: +7.9 (+7.4%)	OM: 0.044 mm → 0.048 mm (Δ +0.004 mm) TM: 0.044 mm → 0.047 mm (Δ +0.003 mm)
Penha et al. (2024)	Oral minoxidil 5 mg vs. topical minoxidil 5%	Men with MPHL	Parallel-arm RCT	90	24 weeks	Frontal scalp: OM: 190.2 (SD 61.2) → 201.0 (SD 61.4) TM: 207.4 (SD 63.7) → 215.5 (SD 58.9) Vertex scalp: OM: 190.3 (SD 55.8) → 201.9 (SD 57.7) TM: 211.6 (SD 67.8) → 216.5 (SD 65.1)	Frontal scalp: OM: +10.8 (+7%) TM: +8.1 (+6.4%) Vertex scalp: OM: +11.6. (+8.7%) TM: +4.9 (+4.9%)	Not reported
Sanabria et al. (2025)	Oral minoxidil 5 mg vs. sublingual minoxidil 5 mg	Men with MPHL	Double-blind RCT	85	24 weeks	OM: 187.1 (SD 46.5) → 208.5 (SD 53.5) SLM: 187.6 (SD 45.1) → 210.0 (SD 55.7)	OM: +21.8 SLM: +24.5	Not reported
Bokhari et al. (2022)	Sublingual minoxidil 0.45 mg, 1.35 mg and 4.05 mg vs. placebo	Men with MPHL and women with FPHL	Double-blind RCT	40	24 weeks	Not reported	Frontal scalp: SLM 0.45 mg: +4 SLM 1.35 mg: +10 SLM 4.05 mg: +38 Vertex scalp: SLM 0.45 mg: +9 SLM 1.35 mg: +26 SLM 4.05 mg: +88	Placebo: -2 µm (SD 1 µm) SLM 0.45 mg: -2 µm (SD 1 µm) SLM 1.35 mg: +3 µm (SD 1 µm) SLM 4.05 mg: +6 µm (SD 1 µm)
Olsen et al. (2002)	Topical minoxidil 5% BID vs. topical minoxidil 2% BID vs. placebo	Men with MPHL	Double-blind RCT	393	48 weeks	Not reported	TM 5%: + 18.6 (12.3%) TM 2%: +12.7 (8.8%) Placebo: +3.9 (2.6%)	Not reported
Olsen et al. (2007)	Minoxidil 5% foam BID vs. placebo	Men aged 18-49 years with MPHL	Double-blind RCT	352	16 weeks (+ 52-week safety extension)	Not reported	MF 5%: +20.9 (+13.4%) Placebo: +4.7 (+3.4%)	Not reported

Abbreviations: BID, twice a day; FPHL, female pattern hair loss; MF, minoxidil foam; MPHL, male pattern hair loss; OM, oral minoxidil; RCT, randomized controlled trial; SD, standard deviation; SLM, sublingual minoxidil; TM, topical minoxidil.

When comparing the 3 routes of administration, each presents distinct advantages and limitations. Topical minoxidil remains the only FDA-approved formulation and has the longest track record, but its effectiveness is hindered by adherence issues and variable follicular sulfotransferase activity. Oral minoxidil provides systemic exposure and is effective even in patients with low follicular SULT1A1 activity, making it valuable in topical non-responders. Its convenience and evidence base are strengths, but its off-label status and the need for monitoring limit widespread adoption. Sublingual minoxidil, although still investigational, is promising and may offer the best balance between efficacy and tolerability by optimising follicular delivery while minimising systemic exposure. However, its long-term safety and comparative effectiveness remain to be established. The Samson Clinical Phase III RCT of sublingual minoxidil in MPHL is expected to provide more robust data on efficacy and safety (Samson, 2025).

Combination approaches may further enhance outcomes in AGA. In a randomised trial which enrolled 450 men with MPHL, the efficacy of topical minoxidil 5% twice daily, finasteride 1 mg and their combination was evaluated over 12 months. Improvement was observed in 94.1% of participants receiving combination therapy, in contrast to 80.5% with finasteride and 59.0% with topical minoxidil alone as rated by global photographic evaluation, indicating superior efficacy of the combination regimen (Hu et al., 2015). In another RCT, weekly microneedling combined with topical minoxidil 5% resulted in a mean hair count increase of 91.4/cm^2^ compared with 22.2/cm^2^ with topical minoxidil monotherapy after 12 weeks (Dhurat et al., 2013). A recent meta-analysis including 6 clinical trials (n = 343) demonstrated that platelet-rich plasma (PRP) combined with minoxidil demonstrated a significant increase in hair density (weighted mean difference = 9.14/cm^2^) and hair shaft diameter (4.72 µm) compared to minoxidil or PRP alone (Xiao et al., 2024). These findings suggest that combining pharmacological and procedural modalities, such as finasteride, microneedling or PRP, with minoxidil may optimise hair growth, particularly in refractory cases of AGA. However, due to the limited number of studies and heterogeneity in protocols, larger and higher-quality RCTs are needed to validate these findings and establish standardised treatment regimens.

The diversification of minoxidil delivery routes supports a more individualised approach to AGA management, allowing therapy to be tailored to patient characteristics, treatment goals and tolerability. Patients with early-stage disease may benefit from topical formulations, while those with inadequate response or poor tolerance may be candidates for systemic therapy. The choice between oral and sublingual administration may eventually be guided by pharmacogenetic profiling of sulfotransferase activity, although this remains investigational.

After decades of limited therapeutic innovation, the refinement of minoxidil delivery routes marks a significant advance in the management of AGA. Ongoing and future studies will be essential to define optimal dosing, confirm long-term safety and determine whether sublingual minoxidil offers a proven advantage over oral therapy. Within the next 5 years, both oral and sublingual minoxidil are expected to become increasingly incorporated into routine clinical practice, broadening the therapeutic armamentarium for AGA and allowing treatment to be tailored more precisely to individual patient needs.

## 10 Limitations

The available evidence on topical oral, and sublingual minoxidil contains several important limitations. Most studies are characterised by small sample sizes, and considerable heterogeneity in study design, populations, dosing regimens, treatment duration, scalp target sites (vertex vs. frontal), endpoints (total vs. terminal hair density, hair fibre diameter, hair weight) and outcome assessment methods (e.g., phototrichogram vs. trichoscopy vs. global photography), which limit inter-trial comparability. Only a few head-to-head RCTs have compared the different administration routes and network meta-analyses rely largely on indirect evidence.

Safety data for low-dose oral and especially sublingual minoxidil remain limited. Cardiovascular monitoring and adverse events are not consistently reported, and long-term follow-up beyond 1 year is rare. For sublingual minoxidil, there is also a lack of pharmacokinetic and pharmacodynamic data.

Future research should focus on large, multicentre RCTs with standardised endpoints for hair density and diameter, well-defined safety monitoring protocols, and dose-response and pharmacokinetic studies, particularly for sublingual formulations. Such trials are necessary to establish optimal dosing, confirm long-term safety and define the comparative efficacy of the different routes of administration.

## 11 Conclusion

Minoxidil is the mainstay of the treatment of AGA in both men and women. With new data emerging from oral and sublingual administration, minoxidil is no longer viewed as a one-size-fits-all therapy. Instead, it is increasingly prescribed in personalised, context-specific ways based on treatment response, tolerability and patient preference.

Topical minoxidil continues to serve as a popular treatment for AGA in men and women. Its favourable safety profile, affordability and ease of access make it a reasonable starting point for most patients. However, a significant proportion of individuals fail to achieve satisfactory hair regrowth. Additionally, local side effects, e.g., irritation can negatively affect adherence, particularly in women.

LDOM has emerged as a viable alternative in such patients. The data supporting LDOM are increasingly robust, with retrospective and prospective studies consistently showing improvements in hair density and high patient satisfaction rates. In spite of a few side effects, including hypertrichosis and mild cardiovascular symptoms, the overall tolerability of LDOM at doses ≤5 mg/day is favourable. Larger double-blind RCTs may help consolidate LDOM’s positioning in the armamentarium of treatments for AGA.

Preliminary data suggest that sublingual minoxidil, though still investigational, may be a promising alternative. The limited but encouraging data have demonstrated comparable efficacy to oral minoxidil with potentially fewer systemic side effects. If future larger clinical trials confirm its efficacy and safety, sublingual minoxidil may offer a compelling alternative, particularly for patients with cardiovascular comorbidities.

It is worth reiterating that, unlike topical minoxidil, the oral and sublingual formulations are not FDA-approved and only prescribed off-label.
